# Correction: NGS read classification using AI

**DOI:** 10.1371/journal.pone.0301793

**Published:** 2024-04-01

**Authors:** Benjamin Voigt, Oliver Fischer, Christian Krumnow, Christian Herta, Piotr Wojciech Dabrowski

An additional affiliation is missing for the first author. Benjamin Voigt is also affiliated with the Institute of Pathology, Charité - Universitätsmedizin Berlin, corporate member of Freie Universität Berlin and Humboldt University of Berlin, Berlin, Germany.
