# Supplementary material for: NGS read classification using AI
Source: PLoS One. 2021 Dec 22;16(12):e0261548. doi: 10.1371/journal.pone.0261548 (PMC8694450; doi:10.1371/journal.pone.0261548)
Supplement: S1 Appendix — (PDF) [file pone.0261548.s001.pdf]

S1 Appendix. Classification with variable sequence length. As the training of both classifiers was performed with patches of size 100 only we investigate the dependence of the classification accuracy of the taxonomic classification model on the length of the input sequence $\mathbf{x}$. For this we generated additional data sets based on 10% of the sequences contained in the test set of the training data for taxonomic classification. For the generation of new data sets with patches we use the original full sequences and a sliding window with varying size $100 + \Delta L$ where ΔL is drawn independently for each window from an uniform distribution. We consider the four cases $\Delta L = 0$, $\Delta L \sim [-10, 10]$, $\Delta L \sim [-25, 25]$ and $\Delta L \sim [-50, 50]$ and show in Fig the accuracy of the taxonomic classification. We find that the accuracy decreases only very weakly with increasing fluctuations of the patch size and even far from the training setting $\Delta L = 0$ we find that the taxonomic classification performs reliably underlining the fact that the strict patch size limitation in the training does not limit the classification model. The dataset is deposited at zenodo [51].

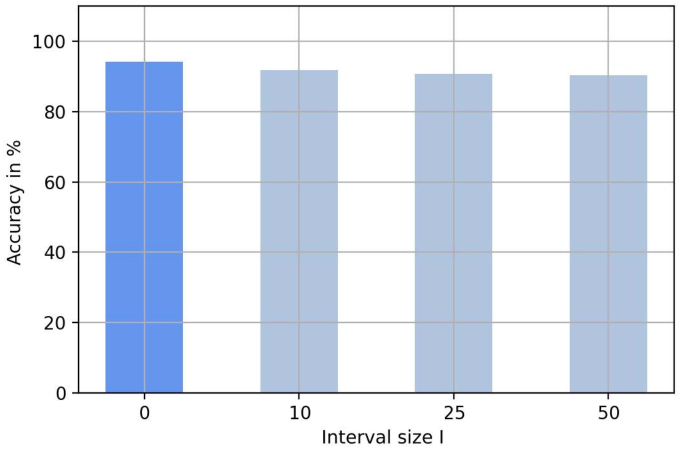

S1 Figure Species classification with sequences of variable length. The patches in the corresponding data set are of size $100 + \Delta L$ with ΔL drawn iid. from $[-I, I]$.

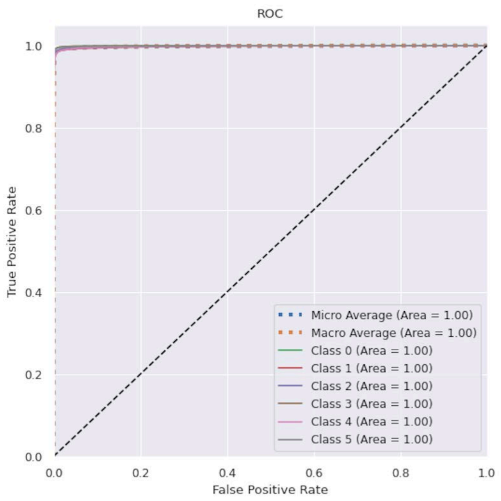

S2 Figure Frame classifications' ROC curve. ROC for the frame classification calculated on the test dataset.
