# Supplementary material for: NGS read classification using AI
Source: PLoS One. 2021 Dec 22;16(12):e0261548. doi: 10.1371/journal.pone.0261548 (PMC8694450; doi:10.1371/journal.pone.0261548)
Supplement: S2 Appendix — (PDF) [file pone.0261548.s002.pdf]

**S2 Appendix. Frame classification with realistic NGS reads.** The training of the both classifiers was performed with ideal data. In realistic NGS applications, the provided reads however contain sequencing errors such as the misread of individual base pairs (single nucleotide polymorphism) or the insertion of additional or deletion of bases. In order to test the performance of our setup in more realistic settings we generate test data with artificially introduced sequencing errors and investigate the performance of the frame classification.

The test data was generated using art_illumina [52] and is based on the test set of [41]. We consider three settings: As art_illumina natively only supports reads up to length 250, in the first setting $T_{E1}$ we cut the patches down to a length of 249 bases and generate simulated reads of the same length. By requiring the input length as output length we ensure that the simulated reads lies in the same reading frame as the input sequence (up to reverse complementation that is randomly applied by art_illumina and reverted by us in the data generation process). In the second setting $T_{E2}$ we generated a 300 base error profile from SRR run SRR3022221 (yielding higher error rates across the entire read length than the default art_illumina 250 base error profile). As in realistic reads, errors tend to accumulate at the end of the read. We consider this in the third setting $T_{E3}$, where we use the simulated reads of the second second setting but use only the starting 249 base-pairs. The resulting three sets are deposited at zenodo [53].

**Table 1. Overall and inner-class accuracies of $P_{frame}$ on the test set [41] with ($T_{E1}$, $T_{E2}$, $T_{E3}$) and without errors ($T$).**

| | $T$ | $T_{E1}$ | $T_{E2}$ | $T_{E1}$ |
|---|---|---|---|---|
| Overall | 0.9817 | 0.9345 | 0.9098 | 0.9247 |
| Frame 0 | 0.9893 | 0.9611 | 0.9652 | 0.9567 |
| Frame 1 | 0.9745 | 0.9126 | 0.8798 | 0.8997 |
| Frame 2 | 0.9773 | 0.9264 | 0.8976 | 0.9157 |
| Frame 3 | 0.9896 | 0.9464 | 0.9219 | 0.9377 |
| Frame 4 | 0.9729 | 0.9213 | 0.8851 | 0.9094 |
| Frame 5 | 0.9866 | 0.9388 | 0.9095 | 0.9292 |

The prediction accuracy of the frame classification model $P_{frame}$ on the different settings and the plain test set is summarized in Table 1. Overall the accuracy of the frame classification is only weakly reduced by errors. We note that a simulated read might contain no errors and match the original sequence to put the results into perspective. In the three settings $T_{E1}$, $T_{E2}$ and $T_{E3}$ correspondingly 36%, 6% and 26% of all simulated reads contain no error while all other sequences include at least one simulated sequencing error. Hence, the vast majority of erroneous sequences are correctly classified. In addition, standard error-reduction practices in real-life applications, like trimming of a simulated NGS read, improve the accuracy here as well. Compare the results in Table 2 for column $T_{E2}$ and column $T_{E3}$.

Therefore, we conclude that although our frame classification model was trained on perfect data, it extrapolates well to more realistic settings and displays robustness under sequencing error in the inputs.
